# The Viscoelastic Properties of the Fungal Cell Wall Allow Traffic of AmBisome as Intact Liposome Vesicles

**DOI:** 10.1128/mBio.02383-17

**Published:** 2018-02-06

**Authors:** Louise Walker, Prashant Sood, Megan D. Lenardon, Gillian Milne, Jon Olson, Gerard Jensen, Julie Wolf, Arturo Casadevall, Jill Adler-Moore, Neil A. R. Gow

**Affiliations:** a Aberdeen Fungal Group, Institute of Medical Sciences, Medical Research Council Centre for Medical Mycology at the University of Aberdeen, Foresterhill, Aberdeen, United Kingdom; b Gilead Sciences Inc., San Dimas, California, USA; c Department of Microbiology and Immunology, Albert Einstein College of Medicine, Bronx, New York, USA; d Cal Poly Pomona, Pomona, California, USA; e School of Biotechnology and Biomolecular Sciences, University of New South Wales, Sydney, NSW, Australia; Duke University

**Keywords:** amphotericin B, antifungal drug, cell membranes, fungal cell wall, mannoproteins

## Abstract

The fungal cell wall is a critically important structure that represents a permeability barrier and protective shield. We probed *Candida albicans* and *Cryptococcus neoformans* with liposomes containing amphotericin B (AmBisome), with or without 15-nm colloidal gold particles. The liposomes have a diameter of 60 to 80 nm, and yet their mode of action requires them to penetrate the fungal cell wall to deliver amphotericin B to the cell membrane, where it binds to ergosterol. Surprisingly, using cryofixation techniques with electron microscopy, we observed that the liposomes remained intact during transit through the cell wall of both yeast species, even though the predicted porosity of the cell wall (pore size, ~5.8 nm) is theoretically too small to allow these liposomes to pass through intact. *C. albicans* mutants with altered cell wall thickness and composition were similar in both their *in vitro* AmBisome susceptibility and the ability of liposomes to penetrate the cell wall. AmBisome exposed to ergosterol-deficient *C. albicans* failed to penetrate beyond the mannoprotein-rich outer cell wall layer. Melanization of *C. neoformans* and the absence of amphotericin B in the liposomes were also associated with a significant reduction in liposome penetration. Therefore, AmBisome can reach cell membranes intact, implying that fungal cell wall viscoelastic properties are permissive to vesicular structures. The fact that AmBisome can transit through chemically diverse cell wall matrices when these liposomes are larger than the theoretical cell wall porosity suggests that the wall is capable of rapid remodeling, which may also be the mechanism for release of extracellular vesicles.

## IMPORTANCE

AmBisome is a broad-spectrum fungicidal antifungal agent in which the hydrophobic polyene antibiotic amphotericin B is packaged within a 60- to 80-nm liposome. The mode of action involves perturbation of the fungal cell membrane by selectively binding to ergosterol, thereby disrupting membrane function. We report that the AmBisome liposome transits through the cell walls of both *Candida albicans* and *Cryptococcus neoformans* intact, despite the fact that the liposome is larger than the theoretical cell wall porosity. This implies that the cell wall has deformable, viscoelastic properties that are permissive to transwall vesicular traffic. These observations help explain the low toxicity of AmBisome, which can deliver its payload directly to the cell membrane without unloading the polyene in the cell wall. In addition, these findings suggest that extracellular vesicles may also be able to pass through the cell wall to deliver soluble and membrane-bound effectors and other molecules to the extracellular space.

## INTRODUCTION

The fungal cell wall is a complex matrix of polysaccharides and proteins that are almost universally absent in mammalian cells. For this reason, they are excellent specific targets for existing antifungal drugs, such as the echinocandins, and the focus of much research looking for novel antifungal agents. For most fungi, the wall is a layered structure, with the inner cell wall being composed of a core, largely conserved laminate of β-glucans and chitin that establishes the strength and physical shape of the wall and the outer wall being more species specific in nature (1, 2). In *Candida albicans*, the outer cell wall is enriched with a fibrillar layer of highly glycosylated mannoproteins which have important roles in defining physical properties such as hydrophobicity and porosity as well as adhesive and immunologic characteristics (3, 4). In *Aspergillus fumigatus*, the outer wall has less protein but includes two bioactive polysaccharides, galactomannan and galactosaminoglycan, while in *Cryptococcus* species, the outer wall is surrounded by a thick capsule composed of glucuronoxylomannan (GXM) and galactoxylomannan (GalXM). The *Candida* mannoprotein fibrillar layer and the *Cryptococcus* capsule are also protective barriers against host enzymes and microbicides and act to impair macrophage phagocytosis and recognition of the underlying β-1,3-glucan layer that is a strong activator of myeloid cell secretion of inflammatory cytokines (2, 5). The fungal cell wall is absolutely essential for the viability and ecology of all fungi, and as such, it is one of the most complex and highly regulated structures in the microbial world.

Recent studies have focused on the composition, as well as the biochemical and immunologic aspects, of fungal cell walls; however, new imaging and analytic technology platforms are beginning to reveal novel biophysical and structural aspects of the cell wall that are likely to prove critical to our understanding of these structures. Sample preparation techniques for transmission electron microscopy (TEM), such as high-pressure freezing followed by freeze-substitution (HPF-FS), have enabled us to visualize unprecedented architectural details such as the structure of the *Candida* mannoprotein fibrils (6, 7) and *Cryptococcus* capsule (1, 8) and the presence of membrane vesicles within the cell wall matrix (9, 10). The presence of such vesicles begs an explanation as to how such large vesicles transit from the membrane through the wall to the external fluid around a cell.

In *C. albicans* and other yeasts, including *Saccharomyces cerevisiae*, a number of approaches have been taken to measure the cell wall porosity. Studies using the high-molecular-weight polysaccharide inulin estimated that the interspace volume of the yeast wall is 23 to 33% of the total volume, suggesting that the wall is an open, porous structure (11). Several studies indicate that the outer mannoprotein layer of yeast determines wall porosity, and porosity assays based on measurement of cell lysis due to DEAE-dextran, poly-l-lysine, and glucanase suggest that molecules with a hydrodynamic radius up to 5.8 nm, equivalent to a molecular radius (Mr) of 400,000 Da, are able to permeate the wall (12, 13). It is also known that the secretome, which contains many large glycoproteins such as invertase and other enzymes (up to 200,000 Da), is able to pass through the yeast cell wall (14).

AmBisome, the first antifungal agent licensed for the treatment of systemic fungal infections (15, 16), must pass through the fungal cell wall to reach the fungal cell membrane, where it binds with ergosterol to produce membrane leakiness (17). It is a polyene macrolide antibiotic produced by the bacterium *Streptomyces nodosus* and is one of very few broad-spectrum chemotherapeutic agents available for the treatment of systemic fungal infections. A side effect of treatments using native amphotericin B as an emulsion with deoxycholate is an associated nephrotoxicity that usually requires specific management using diuretics (18–21). Subsequently, a variety of lipid formulations have been devised that significantly mitigate the unwanted clinical side effects of amphotericin B treatment (21). Of the three lipid formulations in clinical practice, AmBisome is the least toxic (22, 23) and is widely used as a treatment of choice, in particular in cases where empirical treatment is required for suspected fungal infection and the agent of disease is not known (24). Some lipid formulations of amphotericin B present the polyene macrolide in a carrier planar lipid sheet or fragment, but AmBisome is the only lipid carrier in which the antibiotic is encapsulated within the liposome membrane bilayer of an intact liposome (17, 18, 23, 25). AmBisome is effective in the treatment of a wide range of fungal diseases caused by *Candida*, *Aspergillus*, and *Cryptococcus* species and by *Mucorales* and other rare pathogens (24).

The estimates of cell wall porosity all suggest that there should be no porosity barrier to free amphotericin B (molecular weight, 923.49) but that an AmBisome liposome, or a cell wall vesicle, would be too large to freely permeate the cell wall. For these reasons, we wondered whether AmBisome would be able to transit the cell wall intact or whether, as might be predicted, it would dissociate within the wall prior to delivering amphotericin B to the cell membrane. We show here that the liposomes containing AmBisome pass through the cell wall layers of both *C. albicans* and *Cryptococcus neoformans* intact and that the intact AmBisome can deliver inelastic 15-nm colloidal gold particles, which are above the ~6-nm theoretical hydrodynamic radius of freely permeating molecules, through the cell wall. These observations suggest a new paradigm for how molecular trafficking through the fungal cell wall is regulated and suggest that AmBisome could be used as a flexible carrier for the delivery of a wide variety of reagents through a wide range of nonporous cell walls. Furthermore, the results have important implications for our understanding of cell wall rigidity and porosity and the mechanisms by which extracellular vesicles are released by fungal cells into the surrounding space.

## RESULTS

### AmBisome transits intact through the *C. albicans* and *C. neoformans* cell wall.

**FIG 1:**
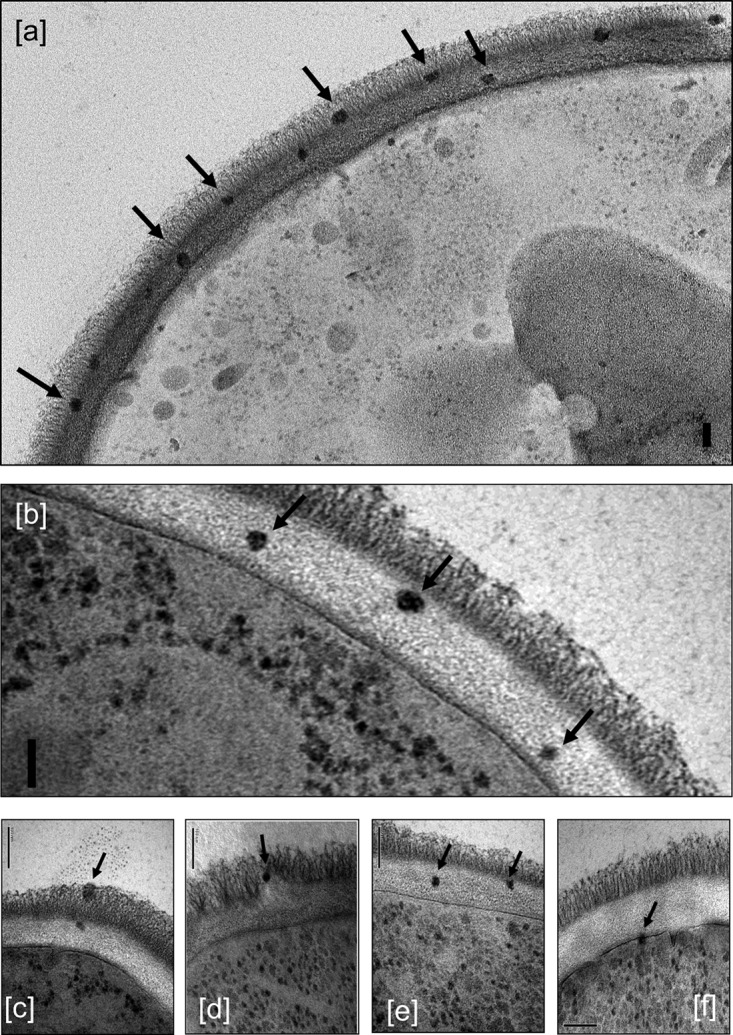
TEM images of *C. albicans* SC5314 incubated with 12 µg/ml AmBisome, showing intact liposomes in the outer (a, c, and d) and inner (a, b, c, and e) cell wall and at the cell membrane (f), indicated by arrows. The granular particles in the cytoplasm are ribosomes, not liposomes. Bars, 100 nm.

Electron-dense, sphere-shaped AmBisome liposomes could be resolved throughout the inner and outer cell wall layers of *C. albicans* using freeze-substitution transmission electron microscopy (Fig. 1). In section, these particles were 20 to 60 nm in most sections, smaller on average than the diameter of the native liposome, perhaps due to shrinkage occurring during sample preparation and because some sections were not medial to the liposome. In tomograms of the *C. albicans* wall, intact liposomes could be readily seen in the inner and outer cell wall layers (see Movies S1 and S2 in the supplemental material). The tomograms, which capture multiple images, displayed full-size liposomes. Liposome particles were also seen intact at the surface of the plasma membrane but not within the cytoplasm of the cell. No such particles were observed in any liposome controls lacking AmBisome (Fig. S1a) or in cells that were treated with 12 μg/ml amphotericin B deoxycholate preparations (Fig. S1b). No accumulation of AmBisome was observed at the outer cell wall surface or at the base of the microfibrillar layer.

10.1128/mBio.02383-17.3 MOVIE S1  Tomogram showing AmBisome liposomes distributed throughout the inner and outer cell wall layers of *C. albicans*. Download MOVIE S1, AVI file, 16 MB. Copyright © 2018 Walker et al. 2018 Walker et al. This content is distributed under the terms of the Creative Commons Attribution 4.0 International license.

10.1128/mBio.02383-17.4 MOVIE S2  Tomogram showing AmBisome liposomes distributed throughout the inner and outer cell wall layers of *C. albicans*. Download MOVIE S2, AVI file, 16 MB. Copyright © 2018 Walker et al. 2018 Walker et al. This content is distributed under the terms of the Creative Commons Attribution 4.0 International license.

10.1128/mBio.02383-17.2 FIG S1  Control TEMs of *C. albicans* cell walls with no AmBisome (a) and 12 μg/ml amphotericin deoxycholate (b), showing the absence of any liposome-like features in the cell walls in the absence of AmBisome. Bars, 100 nm. Download FIG S1, TIF file, 2 MB. Copyright © 2018 Walker et al. 2018 Walker et al. This content is distributed under the terms of the Creative Commons Attribution 4.0 International license.

### AmBisome carries gold particles to the cell membrane.

**FIG 2:**
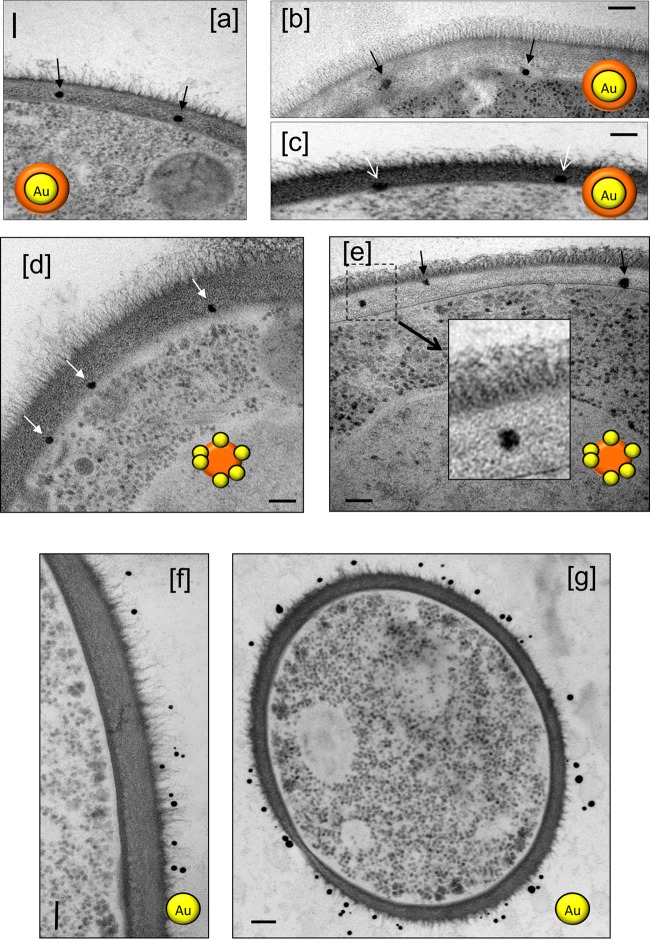
TEMs of cell walls of wild-type *C. albicans* SC5314 cells revealing AmBisome liposomes encapsulating 15-nm-diameter colloidal gold particles (a to c) or AmBisome liposomal membrane studded with 1.6-nm gold particles (d and e). Control gold unencapsulated particles failing to enter the outer or inner cell walls are shown in panels f and g. Bars, 100 nm. The cartoon icons with orange spheres represent AmBisome liposomes, and the gold spheres represent 15-nm or 1.6-nm gold particles that were either encapsulated inside the liposome or present within the liposomal membrane, respectively. These liposomal reagents were imaged in the indicated panels.

**FIG 3:**
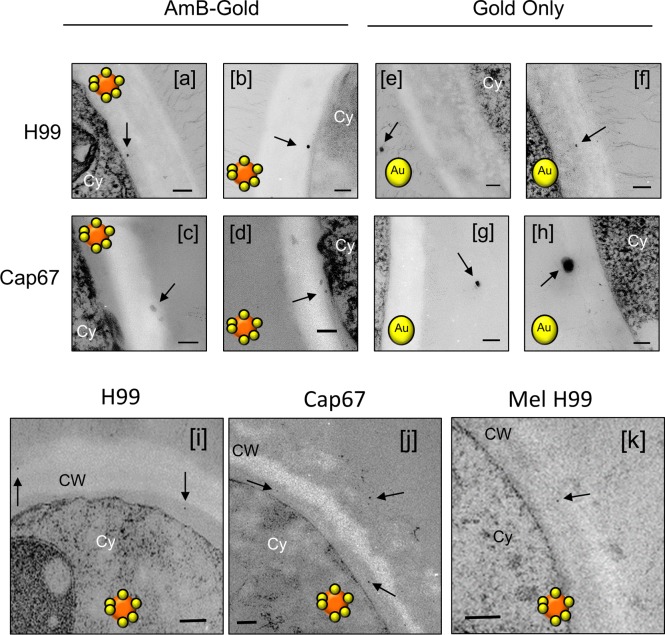
Distribution of AmBisome-encapsulated gold particles (a to d and i to k) or gold-only unencapsulated 1.4-nm gold particles (e to h) relative to the cell wall and capsule of wild-type (H99) *C. neoformans*, an acapsular mutant (Cap67), and a melanin-deficient mutant (Mel H99). Bars, 100 nm. Cy, cytoplasm; CW, cell wall; Ca, capsule. The colored cartoon icons are liposomal reagents as described in the legend to Fig. 1.

Because the AmBisome liposomes are larger than the estimates of 5.8 nm for the maximum hydrodynamic radius of particles that could diffuse through the cell wall, we wished to test whether the liposomes deformed as they passed through the sieve of polysaccharides in the cell wall. We used liposomes that had encapsulated 15-nm-diameter colloidal gold particles within the liposome lumen and liposomes that had smaller, 1.6-nm gold particles associated with the outside of the liposome bilayer. Remarkably, both types of gold-labeled liposomal particles could be observed throughout the cell walls of *C. albicans* and *C. neoformans* (Fig. 2a to e and 3). Since it is not feasible that the 15-nm gold particles could deform as they passed through the mesh of the cell wall during transit, this implies that the liposomes displaced polysaccharide chains of the cell wall around them as they moved through the wall. As controls, 15-nm gold particles, devoid of a liposomal carrier, were also used. These were unable to enter either the outer or inner cell wall layers of *C. albicans* (Fig. 2f and g) or the capsule of *C. neoformans* (not shown). AmBisome therefore facilitated the delivery of the gold particles through the mass of the *C. albicans* cell wall. Images of both encapsulated *C. neoformans* (Fig. 3a, b, e, f, and i) and an acapsular mutant of *C. neoformans* (Fig. 3c, d, g, h, and j) also showed liposomes within the capsule layer and within the subcapsular inner cell wall. Therefore, the AmBisome liposomes could penetrate the cell wall and capsule layers, apparently intact, of two phylogenetically distant fungal species, which are known to differ in cell wall architecture and composition.

### Liposomes lacking amphotericin B fail to enter the inner cell wall.

**FIG 4:**
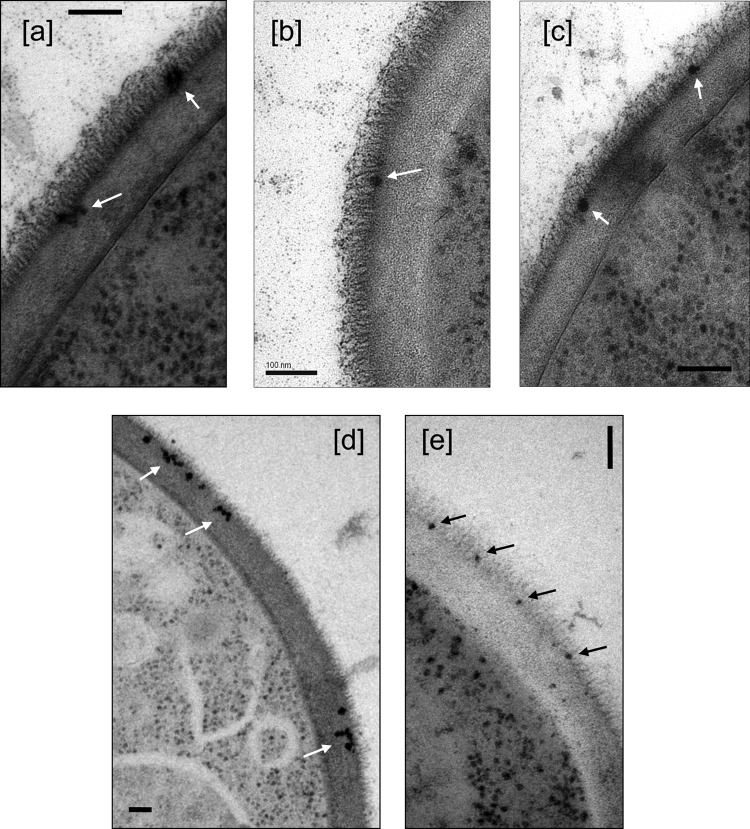
Liposomes with no incorporated amphotericin B (a to c) and an *erg3-1* mutant (d) and *erg11* mutant (e) of *C. albicans* with AmBisome, both showing a deficiency in entering the inner cell wall layer. Bars, 100 nm.

When liposomes that had the same lipid composition as AmBisome but were devoid of amphotericin B were used, they were observed at the base of the fibrils of the outer cell wall of *C. albicans*, but they did not, or very rarely, entered the inner β-glucan–chitin layer (Fig. 4a to c). For *C. neoformans*, liposomes containing amphotericin B were observed embedded inside the fungal cell wall, often in close proximity to the plasma membrane, whereas liposomes without amphotericin B were excluded from entering the cell wall or were maintained at the exterior cell wall perimeter (not shown). Melanized *C. neoformans* cells, which have a considerably less porous cell wall than nonmelanized cells (26), were more resistant to amphotericin B-liposome transit through the cell wall, with a few liposomes transiting partially through the wall and no membrane-adjacent liposomes observed (Fig. 3i and k).

### Cell wall transit of AmBisome is altered in ergosterol-deficient *Candida* mutants.

We next tested whether the transit of the liposomes would be altered if they were exposed to ergosterol-deficient *Candida* mutants. *C. albicans erg3-1* and *erg11* null mutants had significantly elevated MICs for AmBisome (4 and >16 µg/ml, respectively), compared to 0.5 µg/ml for the wild-type parent strain SC5314. When AmBisome was incubated with the *erg3-1 or erg11* mutant, the liposomes were observed to concentrate at the junction between the outer fibrillar and inner cell wall layers (Fig. 4d and e). Similarly, when an *erg3/erg11* double null mutant of *Candida tropicalis* was used, the AmBisome MIC increased from 1 to 16 μg/ml and the liposomes again failed to enter the inner cell wall layer and remained concentrated at the base of the outer cell wall microfibrils (images not shown).

### AmBisome affects cell wall porosity.

**FIG 5:**
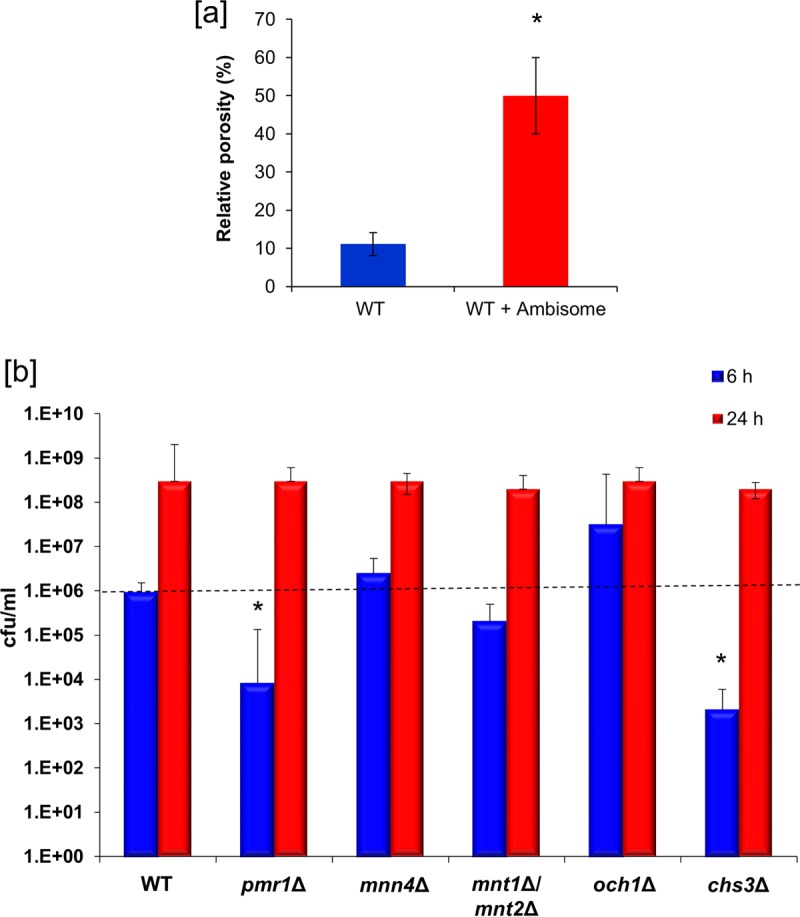
(a) Assay of the relative porosity of the cell wall of wild-type (WT) *C. albicans* SC5314 in the presence and absence of 12 µg/ml AmBisome. Error bars are standard deviations (*n* = 3). (b) Relative killing of *C. albicans* CAI-4 (wild type) and a range of null mutants with deletions in cell wall synthesis genes at a low concentration (0.5 µg/ml) of AmBisome to show relative inhibition due to various perturbations in cell wall composition after 6 h and 24 h. The error bars are standard deviations (*n* = 3); asterisks show significant differences from control (*P* > 0.05).

Since AmBisome liposomes seemed to be able to transit through cell walls with a porosity that was predicted to exclude them, we next tested whether AmBisome affected the measured cell wall porosity of *C. albicans* using a standard porosity assay based on assessing the relative speed of penetration of two detergents with different molecular weights and their ability to cause cell lysis. In this assay, a short, 1-h exposure was used, at which time no measurable decrease in cell viability was observed by plating of viable cells on yeast extract-peptone-dextrose (YPD) agar, but the apparent relative porosity of the cell wall increased 5 times in the presence of AmBisome (Fig. 5a).

### Mutations in cell walls of *C. albicans* do not alter AmBisome sensitivity.

We then examined a range of *C. albicans* cell wall mutants that we knew were altered in cell wall thickness or in their chitin or mannan content and assessed whether these mutations altered their sensitivity to AmBisome and the transit of AmBisome through the cell wall (images not shown). The *mnt1*Δ*/mnt2*Δ, *pmr1*Δ, and *och1*Δ glycosylation mutants had reduced thickness in the outer cell wall microfibrillar layer, and the *mnt1*Δ/*mnt2*Δ and *pmr1*Δ mutants also had a thinner inner layer (Table 1). The *och1*Δ mutant is known to activate the Mkc1-dependent cell wall salvage mitogen-activated protein kinase (MAPK) pathway, resulting in a thickened cell wall due to the upregulation of chitin synthesis (27) (Table 1). The *chs3* mutant lacks 80 to 90% of wild-type cell wall chitin. None of these mutants had major differences in their 50% inhibitory concentrations (IC_50_s) for AmBisome at 24-h and 48-h time points, although the IC_50_ of the *och1*Δ mutant was slightly elevated and the *chs3*Δ mutant had a slightly reduced IC_50_ (Table 1; Fig. 5b). To determine the susceptibility of these cell wall mutants to AmBisome at an earlier time point, the CFU of each mutant after exposure to AmBisome for 6 h and 24 h was determined. At 6 h, the *pmr1* and *chs3* mutants were more susceptible to AmBisome; however, by 24 h there was no difference in susceptibility to AmBisome for any of the mutant strains (Fig. 5b). Therefore, although there were no substantial differences in MIC in the mutants, the mutant with the increased chitin content (*och1*) was slightly more resistant and the *chs3* mutant with reduced chitin content was slightly more sensitive to AmBisome (Fig. 5b). Chitin may be somewhat of a retardant to AmBisome cell wall penetration, given these mild effects on AmBisome MIC. However, based on electron microscopy, there were no obvious differences in AmBisome distribution, as the liposomes were seen transiting throughout the cell wall of each mutant (not shown). The results indicate that AmBisome can penetrate through the cell wall despite significant differences in cell wall composition.

**TABLE 1:** Mean thickness of *C. albicans* cell wall, measured in 10 TEM medial sections, in comparison with sensitivity to AmBisome measured at 24 h and 48 h

Strain	AmBisome IC_50_ (µg/ml) at time:		Cell wall thickness (nm) ± SD^a^	
	24 h	48 h	Outer wall	Inner wall
Wild type	1	2	77 ± 15	76 ± 17
*pmr1*Δ mutant	0.5	1	46 ± 9	56 ± 23
*och1*Δ mutant	2	4	6 ± 0.8	230 ± 29
*mnn4*Δ mutant	0.5	2	79 ± 9	77 ± 23
*mnt1*Δ/*mnt2*Δ mutant	1	1	34 ± 5	50 ± 17
*chs3*Δ mutant	0.25	0.5	81 ± 20	42 ± 9

a The outer wall refers to the mannoprotein fibrillar fringe, and the inner wall refers to the glucan-chitin amorphous layer.

## DISCUSSION

In this study, we used liposomes containing amphotericin B (AmBisome) with or without 15-nm colloidal gold particles to investigate the fate of these vesicles during their interaction with cells of two fungal species that differ in their cell wall structure. To our surprise, liposomes penetrated the cell walls of both *C. albicans* and *C. neoformans*, structures that are often considered rigid and not porous enough for such large particles. This observation has important implications for (i) the mechanism of AmBisome antifungal action, (ii) the potential usefulness of developing liposomes to deliver cargo to fungal cells, and (iii) our views on fungal cell porosity, viscoelastic properties, and extracellular vesicle transport.

AmBisome has been used clinically in the treatment of fungal infections for more than 25 years, and yet the details of exactly how this drug induces cell death remain to be fully elucidated. Although it has long been assumed that the fungicidal properties of amphotericin B are due to its ability to form membrane channels, it has been suggested more recently that amphotericin B can kill yeast cells by binding membrane ergosterol without forming a channel and that channel formation can be a secondary effect that potentiates the toxicity of this polyene (28), which also stimulates the formation of oxygen radicals (29). Whatever the mechanism or mechanisms, this agent is membrane active and therefore must transit the cell wall of the fungus in order to deliver the amphotericin B to the fungal sterol target in the cell membrane.

AmBisome liposomes contain cholesterol, which contributes to the stability of the liposomes. It is included not to promote fusion but to help retain the amphotericin B in the liposomes until the liposomes bind to the fungal cell wall. The liposomes traverse the fungal cell wall until they come into direct contact with ergosterol. Amphotericin B in the liposomes has a 10-fold-higher affinity for ergosterol than cholesterol, resulting in the release of amphotericin B from the liposomes to interact with the ergosterol in the fungal cell membrane.

Fungal cell walls differ substantially in their cell wall composition, and so it is at one level remarkable that amphotericin B in its liposomal formulation is still among the broadest-spectrum agents available to the clinician. AmBisome liposomes are large in relation to the porosity of the meshwork of polysaccharide chains that compose the bulk of the fungal cell wall, and therefore, it is not clear if they have to disassemble in order for the AmBisome liposome to transit the wall to reach the cell membrane. Some current views of the mechanism of action of AmBisome suggest that these liposomes first bind to cell walls and then dissociate, freeing amphotericin B to travel alone through the cell wall to reach the cell membrane and produce its toxic effects (18). Our data show that AmBisome liposomes seem to migrate through the cell wall intact and discharge their toxic payload only when they reach the inner side of the cell wall at the surface of the hydrophobic membrane. Although there are other liposomal amphotericin B formulations, which have the same or different lipid components, these formulations do not have AmBisome’s efficacy and decreased toxicity profile (22, 23, 30), and thus, the observations reported here cannot be extrapolated to these other liposomal amphotericin B formulations.

**FIG 6:**
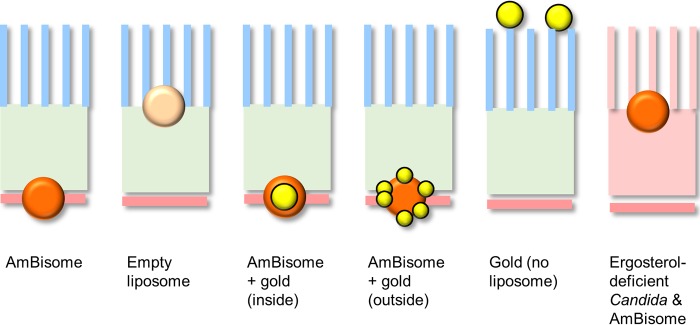
Cartoon summarizing the patterns of distribution of AmBisome liposomes and liposomes carrying gold particles. The outer mannoprotein layer of the cell wall of *C. albicans* is shown as the blue fork-like structure. The cartoon icons with orange spheres represent AmBisome liposomes, and the gold spheres represent 15-nm or 1.6-nm gold particles that were either encapsulated inside the liposome or present within the liposomal membrane, respectively. The light orange sphere represents a liposome devoid of amphotericin B, and the orange wall of the right hand side represents an ergosterol-deficient mutant.

We have also shown that the ability of intact AmBisome liposomes to reach the membrane seems to require the presence of amphotericin B itself, since liposomes that are devoid of the polyene were not able to transit the inner cell wall layer (Fig. 6). The amphotericin B in AmBisome binds with the cholesterol in the liposome bilayer and spans the entire liposome bilayer, forming a pore. When not in contact with a fungus, the amphotericin B in AmBisome remains associated with the liposome bilayer, resulting in decreased mammalian cell toxicity (20). Thus, because amphotericin B in AmBisome is not hidden within the bilayer, it is allowed to interact with the ergosterol when it comes in close proximity to it since its affinity for ergosterol is 10 times higher than its affinity for cholesterol in the liposome bilayer (23). In mutants of *C. albicans* that were ergosterol deficient, we observed that liposomes accumulated at the base of the microfibrils and did not penetrate further into the cell wall (Fig. 6). This may suggest that some ergosterol resides in the cell wall and acts to mediate the transit of AmBisome through the cell wall. Fungal cell walls are known to contain lipid (31–33), possibly as a result of extracellular vesicle transit, and these vesicles will include ergosterol in their membranes (10).

This mode of action of AmBisome with different fungal species helps to explain its broad antifungal activity since it allows AmBisome to target sites of intracellular or extracellular fungal infection without causing host cell toxicity. The liposome initially binds with the fungal cell wall (15, 34) and releases its amphotericin B at the fungal cell membrane only after it transits intact through the cell wall. Since liposomes were not observed in the cytoplasm, liposome dissociation probably occurs at the plasma membrane. Evidence for the intracellular targeting of AmBisome to *Candida* was observed when AmBisome or free amphotericin B was used to treat *Candida* infection in Langerhans cells. TEM results showed killing of the yeast by both forms of the drug but no damage to the host cell with AmBisome and extensive damage to the host cell with the free drug. Tissue histopathology of extracellular *Aspergillus* infection also has shown minimal tissue damage with AmBisome accompanied by significant reduction of the fungal burden (35).

The finding that liposomes containing colloidal gold particles penetrated the cell wall suggests that this system may be adapted to deliver certain cargoes to fungal cells. For example, nucleic acids and other drugs could be packaged in AmBisome-type liposomes to enhance their delivery to fungal cells. The ability of the liposomes containing colloidal gold to penetrate the cell wall was a surprise because this structure is often perceived as a rigid and tough barrier, with a much smaller porosity than the diameter of the liposomes. However, it is important to remember that some of our notions of rigid cell walls that come from the analysis of cell walls isolated by chemical treatments (e.g., zymosan) are inappropriate because they bear little resemblance to living structures. Instead, our observations imply that the cell wall is a dynamic structure with flexible viscoelastic properties. This view is not surprising if one considers how rapidly yeast cells can bud and transform into hyphae and other tip-growing cell types, actions that show the cell wall as a dynamic structure capable of rapid change. There is precedent that even larger structures can cross the fungal cell wall from the outside, given that bacteria have been reported to invade fungal cells by crossing the cell wall to form endosymbionts (36). The fact that the fungal cell wall can be easily rearranged during morphogenesis combined with observations that it can be easily penetrated by bacteria and liposomes suggests that we should rethink this structure as being more akin to a porous and malleable barrier rather than being a rigid structure with low porosity.

The observation that AmBisome liposomes can traverse the cell wall also has important implications for the mechanism(s) for the release of extracellular vesicles (37). Bacterial (38) and fungal species, including *C. albicans* and *C. neoformans*, have now been shown to produce extracellular vesicles, which often carry virulence-related cargo (10, 39, 40). High-resolution electron microscopy has shown vesicles in the cell wall (31), implying that these are released to the extracellular space after cell wall transit. The fact that liposomes can penetrate into the cell wall provides strong support for the notion that a similar mechanism exists in the reverse direction such that vesicles produced by the fungal cell and released at the level of the cell membrane find their way to the extracellular space by direct cell wall transit.

A major question posed by the penetration of the cell wall by AmBisome liposomes is whether some motive force is required for cell wall transport. The fact that liposomes devoid of amphotericin B do not transit the cell wall indicates that something more than this particular liposomal structure is needed for this phenomenon. It is reasonable to assume that some motive force other than diffusion or Brownian motion will be needed for such large structures to cross the cell wall with directionality. One possibility noted above is that the amphotericin B in AmBisome liposomes binds to ergosterol present in the cell wall and liposomes are then transported inward through an existing motive mechanism for vesicular reshuffling. Further support for a motive mechanism in the cell wall comes from cell wall melanin, since melanin is believed to be synthesized in fungal melanosomes that are then transported to the cell wall (41, 42). Consistent with this, melanin “ghosts” isolated from melanized cells have been reported to contain considerable amounts of lipid (43). Since cell wall-associated melanin is constantly remodeled during fungal cell morphogenesis and budding, there must exist mechanisms for moving melanosomes, which in turn could be appropriated by AmBisome liposomes for cell wall transit. Addressing the mechanism that propels cell wall transit is an important area for future studies.

Only the chitin content of the cell wall had a significant effect on the AmBisome MIC, and only melanin, and to a lesser extent chitin, seemed to act as a retardant to AmBisome transit through the cell wall. The melanized cell wall of *C. neoformans* was considerably less permeable to transit by liposomes containing colloidal gold than that of nonmelanized cells. Fungal cell wall melanization involves a process where melanin granules are covalently cross-linked to polysaccharides, including chitin (44), a process that is expected to increase rigidity. Progressive melanization is also accompanied by reduced porosity (26, 45). The combination of increased cross-linking and reduced porosity, which are not necessarily independent, could make the melanized cell wall less permeable to liposomes and reduce the susceptibility of melanized *C. neoformans* to AmBisome. In this regard, melanized fungal cells are known to be less susceptible to amphotericin B (46).

In summary, colloidal gold liposomal particles, combined with electron microscopy, have the potential to be useful tools for studying the fungal cell wall and manipulating the growth of fungi. Using this approach, we found that AmBisome liposomes, including those carrying colloidal gold particles, can cross the fungal cell wall, and this has important implications for our notions of cell wall structure and mechanisms of AmBisome drug delivery. These observations that liposomes can migrate through the cell wall provide insights into how fungal exosomes may transit through the wall into the extracellular space (37). Like most exciting scientific findings, this observation raises more questions than it answers, which in turn suggest new avenues for inquiry. For example, perturbing the system by changing temperature and liposomal lipid composition, combined with colloidal gold electron microscopy, could produce additional insights to understand this phenomenon. In this regard, fungal cells are susceptible to AmBisome at 35°C or at room temperature but not at 4°C, while free amphotericin B is active across a wide range of temperatures (47). In light of our observations, temperature effects on transport across the cell wall are another variable to be considered in interpreting such findings. Finally, it is possible that liposomes could be engineered to carry nonpermeant drugs, antibodies, or enzymes in the liposome lumen in order to deliver them to the fungal cytosol through the otherwise impermeable fungal cell wall.

## MATERIALS AND METHODS

### Strains, mutants, and growth conditions.

*C. albicans* strains were maintained on Sabouraud dextrose (Sabdex) agar plates (1% [wt/vol] mycological peptone, 4% [wt/vol] glucose, and 2% [wt/vol] agar). Strain SC5314 (serotype A) and the genetically derived CAI-4 were used predominantly in TEM imaging experiments. A range of isogenic mutants (created in the CAI-4 background) with disruptions in their cell wall were also examined, including mutants with defects in outer chain *N*-mannosylation (*och1*Δ, NGY357) (27), *O*-mannosylation (*mnt1*Δ *mnt2*Δ, NGY337) (48), or phosphomannan biosynthesis (*mnn4*Δ, CDH15) (49) or mutants downregulated in glycosylation due to low levels of Mn^2+^ in the Golgi complex (*pmr1*Δ, NGY355) (49). The *chs3*Δ strain was provided by Christine Bulawa (50), and the *C. albicans* and *C. tropicalis erg3*Δ and *erg11*Δ mutants were gifts from Dominique Sanglard (51). *C. albicans* strains were first grown overnight in orbital shake flask cultures at 200 rpm at 30°C in Sabouraud broth (1% [wt/vol] mycological peptone, 4% [wt/vol] glucose) and then inoculated into fresh RPMI 1640 medium for 1 h at 37°C in the presence of 12 µg/ml AmBisome prior to harvesting for TEM. A short exposure to AmBisome of only 1 h was chosen to allow AmBisome to be observed in the early stages of entry into the cell. By this time, there was no measurable effect on cell viability or membrane damage. For the same reasons, AmBisome was added to cells for 1 h prior to the HPF-TEM and porosity assays described below.

### High-pressure freezing–freeze substitution transmission electron microscopy.

*C. albicans* yeast cells were first grown in RPMI 1640 in the presence of 12 µg/ml AmBisome for 1 h, before extensive fungal cell lysis took place, and then pelleted and washed with sterile H_2_O. This super-MIC of AmBisome was chosen in order to be able to image sufficient liposome in single serial sections and tomograms. The AmBisome-treated yeast cells were snap-frozen in liquid nitrogen at high pressure using a Leica Empact high-pressure freezer (Leica, Milton Keynes, United Kingdom). The frozen samples were then fixed in an automatic temperature-controlled Leica AFS freeze substitution system in dried acetone containing 2% (wt/vol) OsO_4_, 1% (wt/vol) uranyl acetate, 1% (vol/vol) methanol, and 5% (vol/vol) water in acetone at −90°C for 48 h (52). Samples were then warmed to −30°C and processed in a Lynx tissue processor with 1:2 acetone-resin and embedding in TAAB812 (TAAB Laboratories, Aldermaston, United Kingdom) epoxy resin. One-hundred-nanometer sections were cut with a Leica Ultracut E microtome and stained with uranyl acetate and lead citrate. Samples were examined using a Philips CM10 transmission microscope (FEI UK Ltd., Cambridge, United Kingdom), and images were captured using a Gatan BioScan 792 camera system (Gatan UK, Abingdon, United Kingdom). The average thicknesses of the inner and outer cell wall layers were measured using ImageJ for >20 measurements for each strain.

For *C. neoformans* studies, *C. neoformans* yeast cells were similarly grown in a defined minimal medium (29.4 mM KH_2_PO_4_, 10 mM MgSO_4_, 13 mM glycine, 15 mM dextrose, and 3 µM thiamine-HCl) at 30°C to mid-exponential phase. Samples were then high-pressure frozen using a Bal-Tec HPM 010 high-pressure freezer (Boeckler Instruments, Tucson, AZ). Frozen samples were transferred to an RMC FS-7500 freeze substitution unit (Boeckler Instruments, Tucson, AZ) and freeze substituted in 2% osmium tetroxide, 1% uranyl acetate, 1% methanol, and 5% water in acetone. They were brought from −90°C to room temperature over 2 to 3 days, rinsed in acetone, and embedded in LX112 epoxy resin (Ladd Inc., Burlington, VT). Ultrathin sections of 70 to 80 nm were cut on a Leica Ultracut UC7 microtome, stained with uranyl acetate followed by lead citrate, and viewed on a JEOL 1200EX transmission electron microscope at 80 kV.

### Electron tomography.

The *C. albicans* specimens prepared as described above for TEM were also employed for tomography. Thicker sections (225 nm) were cut using a Leica UC6 ultramicrotome (Leica Microsystems GmbH) onto 200-mesh copper grids and stained with uranyl acetate and lead citrate. Single-axis tilt series of multiple regions of the *C. albicans* cell wall were captured using a JEM-1400 Plus transmission electron microscope (JEOL Ltd., Japan) set at 120 kV and equipped with a bottom-mounted Gatan SC1000 Orius charge-coupled device (CCD) camera (Gatan, USA). After background correction, adjustment of tilt eccentricity, and image calibration, tilts were recorded at an ×20,000 magnification and a 10,000-pixel maximum, between +68° and −68° at 0.5° intervals set by goniometric control with focus readjustment every 4°. The tilt series were acquired by JEOL TEM Recorder v2.7 and TEM Centre for JEM-1400 Plus (JEOL Ltd., Japan).

### Preparation of liposome reagents.

Hydrogenated soy phosphatidylcholine (HSPC), cholesterol, distearoylphosphatidylglycerol (DSPG), amphotericin B, and alpha-tocopherol were dissolved in a 2:1:0.8:0.4:0.01 molar ratio in a 1:1 mixture of methanol and chloroform (or, for some liposomes, the same formula without amphotericin B). Once all components were dissolved, solvents were removed by evaporation under continuous nitrogen flow. Residual solvent was removed by storing the container containing the material in a desiccator under vacuum for at least 48 h. The dried lipid was hydrated in a buffer containing 9% sucrose and 10 mM succinate at the desired drug concentrations, and the hydrated material was processed through a high-shear homogenizer to form liposomes. For encapsulation of conjugated spherical gold nanoparticles (15-nm methyl polymer-conjugated gold nanoparticles; Nanopartz, Inc., Loveland, CO, USA), the buffer was spiked with the nanoparticles during the beginning of hydration. Nonentrapped gold was removed by centrifugation. For samples prepared with ~1.6-nm gold particles associated with the bilayers, dipalmitoyl phosphatidylethanolamine (DPPE) Nanogold (Nanoprobes, Yaphank, NY) was added to the lipid mixture noted above and included in the preparation of dried lipid. The quantities used reflected up to about 10 gold moieties per liposome particle. Liposomes were then prepared as described above. For both types, samples were confirmed to have a median particle size of ~80 nm by dynamic light scattering. Amphotericin B concentration was confirmed by reversed-phase high-pressure liquid chromatography (HPLC) using a C_18_ column and isocratic elution against acetonitrile-methanol-2.5 mM EDTA (25:50:30 [vol/vol/vol]) and using the USP standard.

### Cell wall permeability assay.

*C. albicans* strains were grown overnight at 200 rpm at 30°C in Sabouraud broth (1% mycological peptone-4% glucose) and then washed with sterile H_2_O and inoculated into fresh RPMI 1640 medium for 1 h at 37°C in the presence of 12 µg/ml AmBisome before carrying out the porosity assay. Measurements of the porosity of the *C. albicans* cell wall used the polycationic polymers DEAE-dextran and poly-l-lysine (PLL). These reagents enhance the permeability of the cell membrane, resulting in the leakage of cytosol and nucleic acids (12, 14). Yeast cells were centrifuged and washed three times with double-distilled water (ddH_2_O), and 1 × 10^8^ cells/ml were resuspended in 10 mM Tris-HCl (pH 7.4) containing 10 µg/ml of PLL (molecular mass, 30 to 70 kDa; Sigma) or 5 µg/ml of DEAE-dextran (molecular mass, 500 kDa; Sigma) and incubated at 30°C for 30 min with shaking at 200 rpm. The release of UV-absorbing compounds was determined by measuring the *A*_260_ of cell-free supernatant. The supernatant from cells in Tris-HCl buffer with no polycationic polymer was used as a control. The relative cell wall porosity was defined as relative cell wall porosity (%) = [(*A*_260_ of DEAE-dextran − *A*_260_ of buffer)/(*A*_260_ of PLL − *A*_260_ of buffer)] × 100.

### Antifungal susceptibility testing.

A modified microtiter dilution assay (CLSI method M27) was used to test for the MIC of AmBisome for the various *C. albicans* cell wall mutants, using the indicator dye alamarBlue. Briefly, the yeast cell count was adjusted to a final concentration of 2 × 10^4^ cells/ml in RPMI-morpholinepropanesulfonic acid (RPMI-MOPS). Aliquots of 100 µl/well of a series of 2-fold dilutions of each drug in RPMI-MOPS (0.08 to 40 µg/ml) were dispensed into triplicate wells of a 96-well flat-bottom microtiter plate, with aliquots (100 µl/well) of the yeast suspension dispensed into each test well and 20 µl of alamarBlue dispensed into each well. The plate was incubated at 35°C for 48 h. The MIC was the lowest concentration of drug showing inhibition of growth based on spectrophotometric readings at 570 to 600 nm on a SpectraMax microplate reader (53).

For *C. albicans* viability assays, stationary-phase yeast cells were inoculated to an optical density at 600 nm (OD_600_) of 0.1 and incubated at 30°C with shaking. At each time point, 25-µl volumes of culture were sampled and serially diluted in sterile ddH_2_O to generate suspensions containing 1 × 10^6^, 1 × 10^5^, 1 × 10^4^, 1,000, 100, and 10 cells/ml. Dilutions were spotted onto YPD agar plates and incubated overnight at 30°C. After incubation, the number of colonies was counted and used to determine the viability of each strain over time.

IC_50_ measurements were determined by broth microdilution testing using the CLSI M27-A3 guidelines. The concentration of AmBisome at which the growth of each strain was inhibited by 50% (IC_50_) in YPD for 24 h at 30°C was calculated from the MIC curves.
