# Supplementary figures and images for: The Viscoelastic Properties of the Fungal Cell Wall Allow Traffic of AmBisome as Intact Liposome Vesicles
Source: mBio. 2018 Feb 6;9(1):e02383-17. doi: 10.1128/mBio.02383-17 (PMC5801470; doi:10.1128/mBio.02383-17)

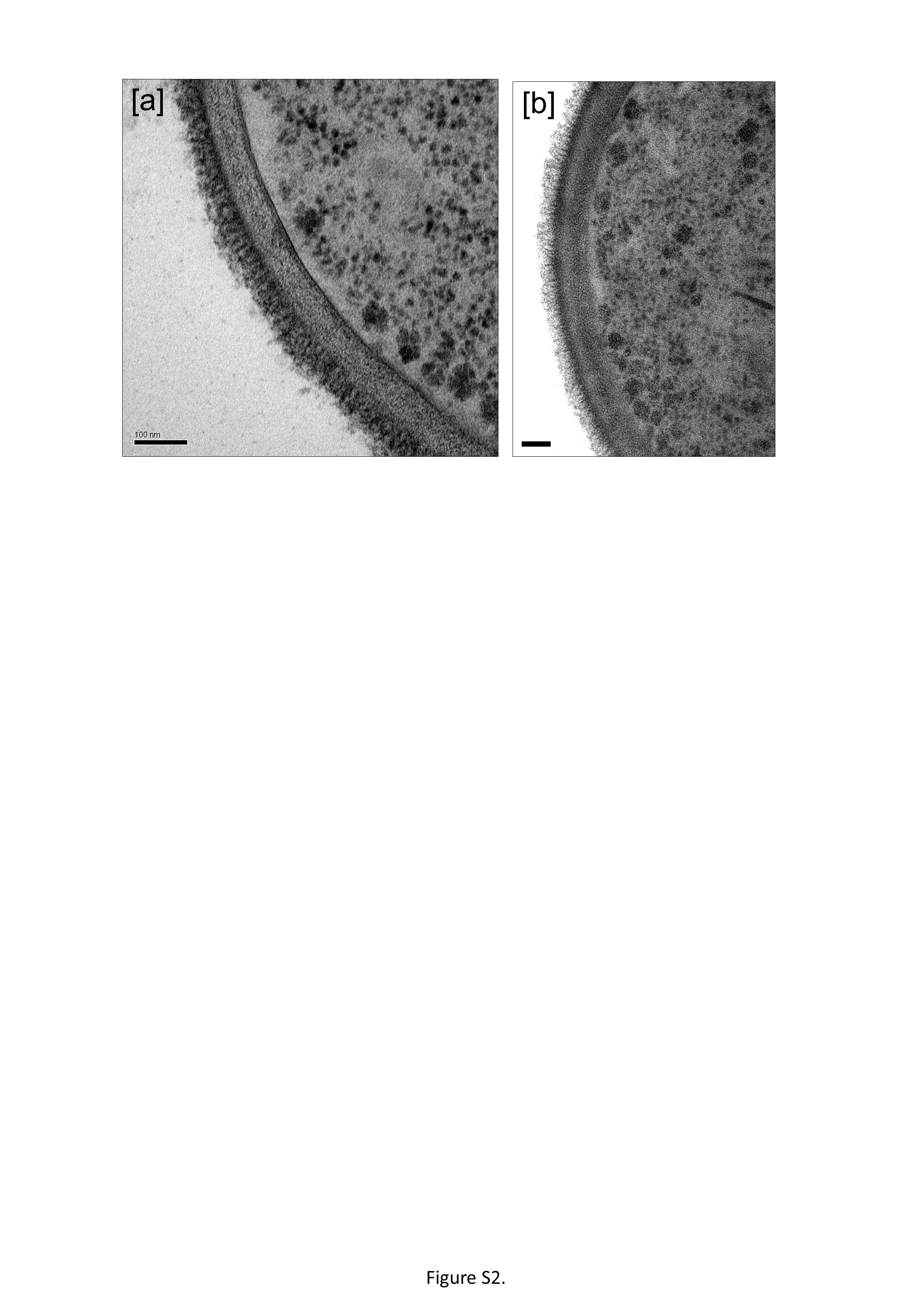

Supplement: FIG S1 [file mbo001183714sf1.tif]
